# Multi-environment evaluation and decision-level analysis of clinical prediction models under dataset shift

**DOI:** 10.3389/fdgth.2026.1874296

**Published:** 2026-10-15

**Authors:** Jorge Robalino-Díaz, Alejandro Cabrera-Andrade, Ana Karina Zambrano, William Villegas-Ch

**Affiliations:** 1 Escuela de Ingeniería en Ciberseguridad, Facultad de Ingeniería y Ciencias Aplicadas, Universidad de Las Américas, Quito, Ecuador; 2 Carrera de Enfermería, Facultad de Ciencias de la Salud, Universidad de las Américas, Quito, Ecuador; 3 Grupo de Bio-Quimioinformática, Universidad de las Américas, Quito, Ecuador; 4 Universidad UTE, Facultad de Ciencias de la Salud Eugenio Espejo, Centro de Investigación Genética y Genómica, Quito, Ecuador

**Keywords:** cross-domain clinical prediction, dataset shift, decision-level analysis, multi-environment learning, probabilistic classification

## Abstract

Clinical machine-learning models are often evaluated within a single dataset, although deployment occurs across heterogeneous institutions and measurement processes. We evaluated Logistic Regression, Random Forest, and Gradient Boosting for ICU hospital-mortality prediction across MIMIC-III Demo and eICU using a 24-h landmark design, explicit feature mappings, and predictors retained only when sufficiently observed in both domains. A decision-level diagnostic analysis examined how predicted-probability distributions interact with a fixed 0.5 threshold. The primary cohort contained 110 MIMIC and 1,324 eICU ICU episodes. In eICU-to-MIMIC transfer, AUC-ROC was 0.598 for Logistic Regression, 0.642 for Random Forest, and 0.613 for Gradient Boosting. Random Forest generated no positive predictions, a threshold failure already visible during internal eICU validation. Logistic Regression retained partial sensitivity with substantial false-positive burden, whereas Gradient Boosting detected only two deaths. These findings show that clinical model evaluation should examine calibration, threshold behavior, feature harmonization, and source-to-target operating-point stability, not only aggregate discrimination.

## 1 Introduction

The use of machine learning models in clinical prediction has grown steadily due to the availability of electronic health records (EHRs) and the ability of these records to capture complex relationships among physiological and biochemical variables. However, much of this development has been evaluated under the assumption of distributional homogeneity, in which training and test data come from the same clinical setting. This condition, while convenient from an experimental perspective, does not reflect the operational reality of healthcare systems, where models must be generalized to different populations, institutions, and protocols. Recent literature indicates that this discrepancy leads to marked performance degradation when models are deployed outside their original domain, particularly in critical tasks such as mortality prediction (1, 2).

The problem is exacerbated in multi-center settings, where heterogeneity between datasets such as MIMIC and eICU introduces differences in variable distribution, event prevalence, and data acquisition processes. These variations directly affect the structure of the models and their generalizability, a phenomenon known as dataset shift. Recent studies have addressed this phenomenon from the perspective of domain adaptation, proposing techniques to align representations across different data sources (3). However, these approaches often focus on improving overall metrics without explicitly analyzing how models make decisions under conditions of distributional misalignment. Additionally, research on feature selection has shown that the stability of selected variables can vary substantially across domains, affecting model consistency (4).

A critical limitation in the evaluation of clinical models arises here: the use of aggregate metrics, such as accuracy or AUC-ROC, does not allow identification of structural flaws in model behavior, especially in detecting rare events. Recent work has indicated that interpretability and understanding of the decision-making process are fundamental for the adoption of models in clinical settings (5), highlighting the need for approaches that go beyond average performance and analyze the relationship between predicted probabilities and final decisions. Furthermore, studies on medical classification have shown that class imbalance and overlap between distributions can materially affect model sensitivity, even when overall metrics suggest good performance (6).

Based on these limitations, this work posits as its central problem the inability of traditional evaluation frameworks to capture the actual behavior of models under conditions of transfer between clinical domains. Specifically, the aim is to answer how the distribution of predicted probabilities changes when a model trained in one environment is evaluated in another, how this variation affects the application of a fixed decision threshold, and consequently, how the model’s ability to detect the positive class is modified. This is integrated into a decision-level diagnostic analysis that enables interpretation of the model’s behavior at the decision level, establishing an explicit connection between probabilistic estimation and the final class assignment.

To address this problem, a multi-environment evaluation protocol is proposed that integrates heterogeneous clinical datasets and defines explicit cross-domain transfer scenarios. This protocol is complemented by a fixed-threshold decision diagnostic focused on the probability distribution $f_{\theta }(x)$ and the application of an unchanged conventional decision threshold, allowing for the characterization of model behavior beyond aggregated metrics. The primary methodology uses variables observed in both clinical domains and restricts feature construction to the first 24 h after ICU admission. A full-episode/full-feature specification is reported separately as a retrospective sensitivity analysis. The methodology includes constructing an integrated dataset from MIMIC and eICU, selecting comparable variables across domains, and evaluating representative models, such as Logistic Regression, Random Forest, and Gradient Boosting, under intra-domain and cross-domain conditions.

The results show that, under the primary 24-h shared-feature specification, all models had limited cross-domain transportability. In Train eICU → Test MIMIC, Random Forest produced no positive predictions at the fixed 0.5 threshold; however, this same threshold failure was already present during internal eICU validation, so it cannot be attributed exclusively to transfer. Logistic Regression retained partial sensitivity with substantial false-positive burden, whereas Gradient Boosting produced three positive predictions, comprising two true positives and one false positive. These exploratory findings indicate that performance degradation is not only manifested in global metrics but also in the structure of the decision-making process itself, highlighting the importance of analyzing the relationship between the probability distribution and the classification threshold. This work empirically characterizes fixed-threshold decision failure under clinical dataset shift, while distinguishing failures already present in the source domain from those amplified during transfer, rather than proposing probability-threshold analysis as a new mathematical construct.

## 2 Literature review

The development of clinical prediction models based on machine learning has evolved rapidly in recent years, driven by the availability of heterogeneous data from EHRs and distributed clinical systems. In this context, algorithms such as Logistic Regression, Random Forest, and boosting methods have demonstrated consistent performance in disease prediction tasks. However, their effectiveness depends largely on data quality, variable selection, and the specific conditions of the clinical environment (1). Recent studies have shown that deep learning models can achieve higher levels of accuracy with complex data. Still, their performance remains conditioned by data distribution and the availability of representative information (2).

One of the main challenges identified in the literature is the generalization of models in heterogeneous clinical settings, where the assumption of independence and identical distribution (i.i.d.) does not hold. This phenomenon, known as domain shift, has been widely documented in medical applications, where variations in equipment, clinical protocols, and population characteristics generate material differences between datasets (7). In this regard, the analysis of distributional shifts has become a central focus of current research, highlighting the need to develop models that maintain their performance outside the training domain.

Empirical evidence indicates that clinical models can experience substantial degradation when evaluated in populations not seen during training. The performance of deep learning models can decrease by 10%–25% when applied across different populations, consistent with the impact of domain shift on generalizability (8). This behavior is attributed to differences in disease prevalence, demographic characteristics, and data collection processes. Furthermore, mitigation strategies, such as domain adaptation and transfer learning, have been observed to offer limited improvements, with performance increases ranging from 5% to 15%, indicating that the problem is not fully resolved by conventional techniques.

In the realm of structured clinical data, integrating multiple information sources introduces an additional layer of complexity. The coexistence of structured and unstructured data, along with heterogeneity across institutions, limits models’ ability to learn cross-domain invariant representations (9). Additionally, data-driven studies, such as MIMIC, have demonstrated that the appropriate selection of variables is critical for maintaining model performance, especially when dimensionality is reduced without substantially affecting predictive capacity (10).

The role of feature selection becomes more relevant in multi-domain scenarios, where the stability of the selected variables directly influences the model’s robustness. The sensitivity of selection algorithms to data variations can affect the model’s consistency across different environments (11). Along these lines, more recent approaches have incorporated advanced techniques, such as hybrid models and methods based on clinical knowledge, which improve interpretability and reduce data redundancy (12, 13).

Another relevant aspect in the literature is external and multicenter validation, which remains limited in many clinical studies. Most models are validated using cross-validation schemes, whereas prospective or multicenter validation is less frequent, thereby limiting the models’ real-world applicability (14). This problem is exacerbated by the lack of standardization in assessment metrics and the definition of clinical outcomes, which hinders comparisons between studies.

Finally, recent literature has begun to explore the relationship between prediction and explainability, highlighting the importance of understanding models’ internal behavior in clinical settings. Although predictive models are effective at identifying patterns, integrating them with interpretable approaches is essential for adoption in medical practice (5). This need becomes critical in scenarios where decisions depend not only on overall performance but also on the model’s ability to respond consistently to data variations.

## 3 Materials and methods

### 3.1 Multi-source data acquisition and domain definition

The study is built upon two primary clinical data sources used for mortality prediction: MIMIC-III Clinical Database Demo v1.4, derived from the MIMIC-III critical care resource (15, 16), and the eICU Collaborative Research Database (17, 18). The multi-environment configuration is defined from these sources using a source_dataset variable and an environment type, thereby allowing distinction between intra-domain and cross-transfer scenarios during evaluation.

MIMIC serves as a reference for a single-center ICU environment, where clinical measurements (vital signs and laboratory tests) follow relatively consistent practices. eICU, on the other hand, integrates data from multiple hospitals and incorporates variability in protocols, instrumentation, and patient profiles. This difference is reflected in the construction of the integrated dataset, where both sources are combined while maintaining the source label, to enable two evaluation configurations: internal validation (training and testing within eICU) and cross-transfer (train eICU → test MIMIC and train MIMIC → test eICU). The unit of analysis was the ICU episode: icustay_id in MIMIC and patientunitstayid in eICU. The binary outcome was in-hospital mortality, operationalized as hospital_expire_flag in MIMIC and actualhospitalmortality in eICU after conversion to a binary label. Records without a resolvable mortality outcome were excluded from supervised modeling.

The prediction target is defined as hospital mortality (target_mortality), derived from the variables available in each clinical source. Integration is performed at the episode/admission level (case_id), preserving the correspondence between records and the output label. This definition allows for direct comparison of model behavior under domain changes while maintaining task consistency.

The operational definition of the multi-domain environment is therefore determined by the two clinical domains with different institutional origins (MIMIC vs. eICU), which are used for intra-domain evaluation and cross-transfer.

Cohort construction followed a deterministic episode-level procedure. MIMIC episodes were extracted from the demo critical-care tables by linking ICU stays to admissions and retaining episodes with an available hospital_expire_flag. The MIMIC-III Demo population was selected from patients who eventually died, although not every included hospital admission ended in death; therefore, its admission-level mortality proportion should not be interpreted as representative of the full MIMIC-III population or of a general ICU population. The local eICU input population was the available patient.csv.gz subset in the authorized local eICU directory, containing 2,520 unique patientunitstayid records. No additional hospital-level or date-range selection rule was applied by the revision scripts; therefore, the eICU input is identified in the archived reproducibility package by source-table names, row counts, file sizes, and SHA-256 checksums rather than by a retrospectively specified sampling rule. Outcomes were obtained from apachePatientResult.actualhospitalmortality; the raw APACHE file contained 3,676 APACHE result rows corresponding to 1,838 unique ICU stays with APACHE results, and these rows were reduced to one APACHE result per patientunitstayid by preferring APACHE IVa over APACHE IV before outcome filtering. Thus, the value 3,676 refers to APACHE result rows, not unique eICU ICU stays. The eICU extract used here is an analyzable subset of the accessible tables rather than a hospital-stratified sample, so hospital representation is not used for inferential claims. The primary 24-h landmark cohort contained 110 MIMIC ICU episodes (33 deaths; 30.0%) from 79 patients and 105 admissions, and 1,324 eICU ICU episodes (117 deaths; 8.8%) from 1,124 patients and 1,277 hospital-system stays, after excluding episodes with identifiable death, ICU discharge, or hospital discharge before completion of the 24-h observation window. Operationally, the primary population is composed of ICU episodes that remained observable in the ICU for at least the first 24 h; episodes with ICU discharge before 24 h were excluded even when the patient may have remained hospitalized, because subsequent non-ICU measurements would not represent the same ICU prediction setting. MIMIC laboratory measurements were joined and aggregated at the ICU-stay level; all retained laboratory rows were verified to fall within their own ICU-specific 24-h window. Patient-grouped internal validation was conducted as a sensitivity analysis, but no hospital-level leave-site-out split was possible with the available MIMIC demo structure.

### 3.2 Data preprocessing and feature construction

Preprocessing is performed independently for each data source, maintaining traceability of each observation through a unique episode identifier (case_id) and its source (source_dataset). In structured clinical datasets (MIMIC and eICU), the variables correspond to time series of physiological and laboratory measurements recorded at different times throughout the patient’s stay (19). The primary analysis uses a landmark design in which measurements are summarized only from the first 24 h after ICU admission. The secondary full-episode construction is retained as a retrospective analysis because it summarized all available episode measurements and can therefore include information recorded after a clinically plausible prediction time. Restricting feature aggregation to the first 24 h reduces the risk of temporal leakage associated with full-stay measurements. Since the time length $T_{i}$ of each episode i is variable, an aggregation process is applied that transforms these series into fixed-dimensional representations.

Let $x_{i,t}^{\left(j\right)}$ be the observed value of the clinical variable j for case i at time t, with $t\in \{1,\ldots ,T_{i}\}$. For each variable, statistical descriptors are constructed that capture both central tendency and intra-episode variability, including the mean (Equation 1), standard deviation (Equation 2), and minimum/maximum values (Equation 3):

$$\mu_{i}^{\left(j\right)}=\frac{1}{T_{i}}\sum_{t=1}^{T_{i}}x_{i,t}^{\left(j\right)}$$ (1)

$$\sigma_{i}^{\left(j\right)}=\sqrt{\frac{1}{T_{i}}\sum_{t=1}^{T_{i}}{\left(x_{i,t}^{\left(j\right)}-\mu_{i}^{\left(j\right)}\right)}^{2}}$$ (2)

$$x_{i,\min}^{\left(j\right)}=\min_{t}x_{i,t}^{\left(j\right)},\;x_{i,\max}^{\left(j\right)}=\max_{t}x_{i,t}^{\left(j\right)}$$ (3)

This set of transformations allows for condensation of the temporal information into a feature vector that preserves the basic dynamics of each variable, regardless of the series length, which is necessary for training supervised models in a homogeneous vector space.

The handling of missing values is addressed using a selection criterion based on the proportion of missing values for each variable. Let $m_{j}$ be the missing value rate of variable j out of the total set of N observations, as defined in Equation 4:

$$m_{j}=\frac{1}{N}\sum_{i=1}^{N}I\left(x_{i}^{\left(j\right)}=\text{NaN}\right)$$ (4)

where I(⋅) is the indicator function. A variable retention threshold is established such that $m_{j}\leq 0.60$, which ensures that each feature included in the model has sufficient statistical support. This filtering prevents the introduction of noise from variables with low completeness and stabilizes training on heterogeneous data.

Once the variables are selected, the features are organized in a common space that includes physiological descriptors observed in both MIMIC and eICU. The primary 24-h shared-feature model used 35 pre-encoding predictors: age, gender, and mean/minimum/maximum descriptors for eleven clinical variable families: heart rate, respiratory rate, systolic blood pressure, diastolic blood pressure, mean blood pressure, glucose, creatinine, white blood cell count, hematocrit, sodium, and blood urea nitrogen. Temperature descriptors were extracted and evaluated for completeness, but excluded from the primary shared-feature matrix because eICU temperature missingness was 91.8%, exceeding the prespecified 60% missingness threshold. The earlier 94.2% temperature missingness estimate was recalculated as 91.8% after applying the corrected 24-h landmark denominator of 1,324 eICU ICU episodes and combining available Celsius values with Fahrenheit values converted as ${(}^{\circ }F-32)/1.8$. After preprocessing, gender was one-hot encoded inside the training pipeline, so the final model matrix could contain more columns than the clinical-variable count. Source-specific predictors were excluded from the primary transfer models, including hospitalid, apachescore, predictedhospitalmortality, and any variable insufficiently observed in one domain. This is essential because predictedhospitalmortality is itself an APACHE-derived mortality estimate in eICU and would constitute a predictor derived for the same endpoint. The secondary 29-variable full-episode/full-feature model is therefore reported only as a retrospective analysis and not as the primary evidence for cross-domain transportability.

The explicit source mappings used for the primary shared-feature extraction are summarized in Table 1. MIMIC laboratory variables were selected by item identifier from LABEVENTS, and vital signs were selected by item identifier from CHARTEVENTS. Creatinine was restricted to the blood/serum/plasma laboratory item represented by MIMIC item ID 50912 and the exact eICU laboratory name creatinine; urinary creatinine and protein/creatinine-ratio measurements were not mapped into the primary creatinine feature family. eICU laboratory variables were selected using exact lower-case lab names in lab, and vital signs were selected from named fields in vitalPeriodic. Temperature was normalized to Celsius for the missingness audit but excluded from modeling.

**Table 1:** Explicit source mappings used for primary 24-h shared-feature extraction.

Feature family	MIMIC source/items	eICU source/field	Restriction or unit handling
Glucose	LABEVENTS: 50809, 50931	lab.labname= glucose	Blood/plasma/serum glucose; mg/dL retained
Creatinine	LABEVENTS: 50912	lab.labname= creatinine	Blood/serum/plasma only; urinary and protein/creatinine-ratio measurements excluded
White blood cell count	LABEVENTS: 51301	lab.labname= wbc x 1000	Blood WBC count; source-aligned count scale retained
Hematocrit	LABEVENTS: 50810, 51221	lab.labname= hct	Blood hematocrit; percent retained
Sodium	LABEVENTS: 50824, 50983	lab.labname= sodium	Blood/plasma/serum sodium; mEq/L retained
Blood urea nitrogen	LABEVENTS: 51006	lab.labname= bun	Blood urea nitrogen; mg/dL retained
Heart rate	CHARTEVENTS: 211, 220045	vitalPeriodic. heartrate	Beats/min retained
Respiratory rate	CHARTEVENTS: 618, 220210	vitalPeriodic. respiration	Breaths/min retained
Systolic blood pressure	CHARTEVENTS: 51, 442, 455, 220050, 220179, 224167, 225309, 227243	vitalPeriodic. systemicsystolic	mmHg retained
Diastolic blood pressure	CHARTEVENTS: 8368, 8440, 8441, 220051, 220180, 224643, 225310, 227242	vitalPeriodic. systemicdiastolic	mmHg retained
Mean arterial pressure	CHARTEVENTS: 52, 456, 220052, 220181, 225312	vitalPeriodic. systemicmean	mmHg retained
Temperature	CHARTEVENTS: Celsius 676, 677, 223762, 226329; Fahrenheit 678, 679, 223761	vitalPeriodic. temperature	Audited only; Fahrenheit converted to Celsius; excluded from primary predictors

### 3.3 Integrated dataset construction and labeling strategy

The integrated dataset is constructed by combining clinical datasets from MIMIC and eICU, ensuring consistency across the feature space defined during preprocessing. Let $D^{\left(M\right)}$ be the set of observations from MIMIC and $D^{\left(E\right)}$ the corresponding set from eICU. The integrated dataset is defined as the union shown in Equation 5:

$$D^{\left(I\right)}=D^{\left(M\right)}\cup D^{\left(E\right)}$$ (5)

where each observation $x_{i}\in D^{\left(I\right)}$ belongs to a common feature space $R^{d}$, aligned between both sources by selecting shared variables.

To preserve the traceability of the data’s origin, a categorical origin variable $s_{i}$ is defined, which identifies the domain to which each observation belongs, as presented in Equation 6:

$$s_{i}=\left\{ \begin{array}{cc} 0,\; & \text{if }x_{i}\in D^{\left(M\right)}\;\\ 1,\; & \text{if }x_{i}\in D^{\left(E\right)}\; \end{array}\right.$$ (6)

This variable allows for explicit distinction between data generation environments and forms the basis for defining intra-domain and cross-transfer evaluation scenarios.

The prediction target is defined as hospital mortality, represented by a binary variable $y_{i}\in \{0,1\}$, constructed from the indicators available in each source, as defined in Equation 7:

$$y_{i}=\left\{ \begin{array}{cc} 1,\; & \text{if the patient has mortality}\;\\ 0,\; & \text{otherwise}\; \end{array}\right.$$ (7)

The integration of the datasets is performed under the condition of consistency in the definition of the target, so that all observations share the same output semantics, regardless of their origin.

Additionally, an environment characterization variable $e_{i}$ is introduced, which captures the differences in the data generation process, as presented in Equation 8:

$$e_{i}=\left\{ \begin{array}{cc} \text{single-center},\; & \text{if }s_{i}=0\;\\ \text{multi-center},\; & \text{if }s_{i}=1\; \end{array}\right.$$ (8)

This distinction enables the incorporation of relevant contextual information for analyzing model behavior, particularly in scenarios where cross-domain variability directly affects generalizability.

The process of building the integrated dataset follows a deterministic sequence that ensures feature alignment, label assignment, and the preservation of each observation’s provenance. This procedure is formalized in Algorithm 1.

Algorithm 1 **Integrated dataset construction for multi-environment learning.**

**Input:**

- $D_{M}$: dataset from MIMIC
- $D_{E}$: dataset from eICU

**Output:**

- $D_{I}$: integrated dataset

1: Align feature space between $D_{M}$ and $D_{E}$

2: Initialize $D_{I}\leftarrow \emptyset$

3: **for** each sample *x* in $D_{M}$ **do**

4:  assign source_dataset ← MIMIC

5:  assign environment_type ← single-center

6:  append *x* to $D_{I}$

7: **end** **for**

8: **for** each sample *x* in $D_{E}$ **do**

9:  assign source_dataset ← eICU

10:  assign environment_type ← multi-center

11:  append *x* to $D_{I}$

12: **end** **for**

13: ensure target variable *y* is defined for all samples

14: return $D_{I}$

This formulation allows for the explicit definition of a multi-domain environment in which observations share a common representation space but differ in their origin and the conditions under which they were generated.

### 3.4 Multi-environment modeling and evaluation protocol

The modeling process is defined on the integrated dataset $D^{\left(I\right)}=\{(x_{i},y_{i},s_{i}){\}}_{i=1}^{N}$, where each observation $x_{i}\in R^{d}$ represents an aligned feature vector, $y_{i}\in \{0,1\}$ corresponds to the target mortality variable, and $s_{i}$ identifies the domain of origin. Three supervised models with different representational capabilities are used: Logistic Regression as a linear model, Random Forest as a decision-tree ensemble, and Gradient Boosting as a sequential additive model oriented toward error minimization.

Model estimation is based on the probabilistic prediction of the target variable, where each model defines a function $f_{\theta }(x_{i})\in [0,1]$ that represents the estimated probability of mortality. The classification decision is obtained by applying a fixed threshold τ, defined in Equation 9:

$${\hat{y}}_{i}=I\left(\;f_{\theta }(x_{i})\geq \tau \right)$$ (9)

where τ=0.5 and I(⋅) is the indicator function. The value τ=0.5 is used as a fixed conventional reference threshold to assess the behavior of an unchanged decision rule; it was not selected in the source validation data and is not presented as an optimized clinical threshold. Because Logistic Regression and Random Forest were trained with balanced class weights, their raw probability outputs should be interpreted as model scores on a probability scale rather than as automatically calibrated estimates of absolute deployment risk. This formulation decouples the probabilistic estimation process from the binary decision, facilitating analysis of the model’s behavior across different distributions.

The experimental protocol is organized into four scenarios that differ in the relationship between the training and test sets. In the internal validation scenario, the model is trained and evaluated solely on eICU data, allowing its behavior to be analyzed under consistent data-distribution conditions. In the integrated cross-validation scenario, the entire dataset $D^{\left(I\right)}$ is used under a stratified five-fold cross-validation scheme, preserving the class proportions in each subset. In the transfer scenarios, the model is trained in one domain and evaluated in another, explicitly defining distributional misalignment conditions.

Evaluation in transfer scenarios is formalized as a cross-domain generalization problem, in which the training and test distributions differ. Let $P_{E}(x,y)$ be the distribution associated with eICU and $P_{M}(x,y)$ the one corresponding to MIMIC. The evaluation condition is established in Equation 10:

$$P_{\text{train}}(x,y)\neq P_{\text{test}}(x,y)$$ (10)

This inequality introduces a domain change that directly affects the model’s generalization capacity, enabling analysis of its behavior under structural and distributional variations. For the eICU internal-validation scenario, a stratified 75%/25% train-test split was used with random_state=42. Integrated cross-validation used StratifiedKFold(n_splits=5, shuffle=True, random_state=42) across the combined MIMIC-eICU clinical dataset. This integrated cross-validation estimates performance when both domains are represented during resampling; it is not interpreted as external validation because observations from both sources contribute to the cross-validation process.

The complete training and evaluation procedure is explicitly described in Algorithm 2, which defines the sequence for scenario selection, model training, prediction generation, and metric calculation. All preprocessing operations were implemented inside a scikit-learn pipeline fitted only on the training split or training fold. Numeric variables were imputed using the training-set median and standardized using the training-set mean and standard deviation. Categorical variables were imputed using the most frequent training-set category and one-hot encoded with unseen categories ignored at test time. Logistic Regression used penalty=l2, C=1.0, max_iter=2000, solver=liblinear, class_weight=balanced, and the default intercept setting. Random Forest used n_estimators=300, criterion=gini, max_depth=None, min_samples_split=2, min_samples_leaf=1, max_features=sqrt, bootstrap=True, class_weight=balanced, and random_state=42. Gradient Boosting used loss=log_loss, learning_rate=0.1, n_estimators=100, subsample=1.0, criterion=friedman_mse, min_samples_split=2, min_samples_leaf=1, max_depth=3, max_features=None, random_state=42, and no class weighting in the primary analysis. No hyperparameter search was performed. The archived revised outputs were generated under Python 3.8.10, scikit-learn 1.3.2, pandas 2.0.3, and NumPy 1.24.3, matching the public environment.yml and requirements.txt files. This algorithm summarizes the experimental process and establishes a direct correspondence between each evaluated configuration and its results.

Algorithm 2 **Multi-environment modeling and evaluation protocol.**

**Input:**

- $D_{I}$: integrated dataset
- Models: {Logistic, RF, GB}

**Output:**

- Performance metrics per scenario

1: Define scenarios:

2:   $S_{1}$: eICU_internal_validation

3:   $S_{2}$: integrated_5fold_cv

4:   $S_{3}$: train_eICU_test_MIMIC

5:  $S_{4}$: train_MIMIC_test_eICU

6: **for** each model in Models **do**

7:  **for** each scenario in $\left\{S_{1},S_{2},S_{3},S_{4}\right\}$ **do**

8:   Split data according to scenario definition

9:   Train model on training set

10:   Predict probabilities on test set

11:   Apply decision threshold τ=0.5

12:   Compute TP, TN, FP, FN

13:   Calculate metrics (Accuracy, Precision, Recall, F1, AUC)

14:  **end** **for**

15: **end** **for**

16: return results

Performance evaluation is based on the confusion matrix, considering the counts of true positives (TP), true negatives (TN), false positives (FP), and false negatives (FN). From these values, the evaluation metrics are defined through Precision (Equation 11), Recall (Equation 12), and F1-score (Equation 13):

$$\text{Precision}=\frac{TP}{TP+FP}$$ (11)

$$\text{Recall}=\frac{TP}{TP+FN}$$ (12)

$$F1=2\cdot \frac{\text{Precision}\cdot \text{Recall}}{\text{Precision}+\text{Recall}}$$ (13)

Additionally, the area under the ROC curve (AUC-ROC) is used as a measure of discriminative capacity independent of the decision threshold, allowing evaluation of class separability across different operating points.

Additional analyses were conducted to evaluate calibration and threshold sensitivity beyond the primary fixed-threshold results. Calibration was summarized using the Brier score, calibration plots, expected calibration error (ECE), and post hoc calibration intercept and slope from a logistic recalibration model of the observed outcome on the logit of the predicted probability. For the primary 24-h analysis, ECE was computed with 10 equal-width probability bins over [0,1] as $\sum_{b}(n_{b}/N)|{\bar{\;p}}_{b}-{\bar{y}}_{b}|$; empty bins contributed zero to the sum. Predicted probabilities were clipped to $\left[10^{-6},1-10^{-6}\right]$ before logit transformation for calibration-intercept and calibration-slope estimation. Average precision was computed as a precision-recall summary for imbalanced outcomes. Finally, post hoc F1-maximizing thresholds were evaluated on the test predictions only as a sensitivity analysis. These optimized thresholds are not proposed as deployable clinical cutoffs because they use test-set outcome information; they are included only to determine whether failure at τ=0.5 reflects complete absence of ranking information or misalignment of the fixed conventional decision threshold. A fully deployable threshold-transportability experiment would require selecting a threshold exclusively within a source-domain validation set or according to prespecified clinical utility, locking it, and then applying it unchanged to the target domain.

A strict shared-feature specification was used as the primary analysis to address imperfect variable equivalence. In this analysis, all predictors that were completely absent in either source were excluded, including eICU-specific identifiers and severity-score variables (hospitalid, apachescore, and predictedhospitalmortality) and pressure-summary variables without equivalent cross-domain extraction. The resulting full-episode sensitivity predictor set contained age, gender, and shared heart-rate, respiratory-rate, glucose, creatinine, white-blood-cell, sodium, and blood-urea-nitrogen summary variables. The 24-h primary predictor set used the corresponding early-window shared variables listed above.

Class imbalance was handled explicitly for Logistic Regression and Random Forest through balanced class weights, whereas Gradient Boosting was evaluated unweighted in the primary analysis because this estimator does not use a class_weight parameter. A sensitivity analysis comparing weighted and unweighted variants was conducted to assess whether class-weighting choices influenced the observed transfer behavior. In Train eICU → Test MIMIC, unweighted Random Forest still generated no positive predictions, and inverse-frequency sample weighting for Gradient Boosting recovered 8 true positives (recall 0.242, F1-score 0.296) but also increased false positives from 1 to 13 and reduced AUC-ROC from 0.613 to 0.573. Thus, class weighting changed the operating point of some models but did not establish robust cross-domain fixed-threshold performance.

The structure of the multi-environment evaluation protocol, including dataset integration, internal validation, and cross-transfer scenarios, is presented in Figure 1, which illustrates the relationships between domains, training and testing paths, and the configurations used in each experimental scenario.

**Figure 1:**
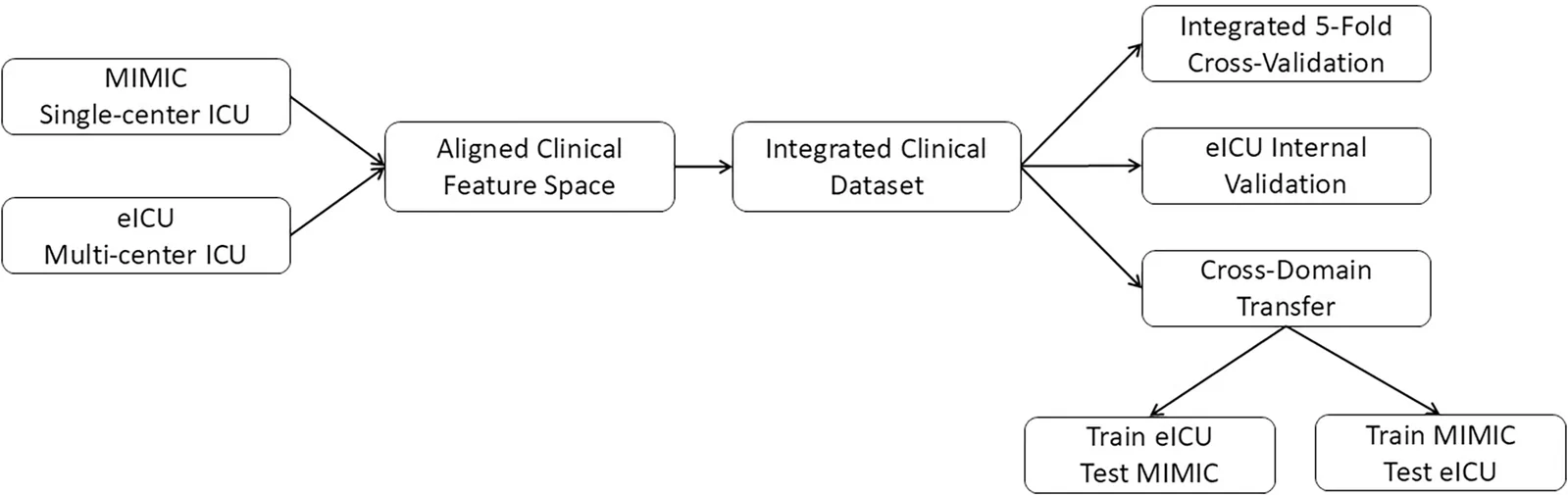
Multi-environment modeling and evaluation protocol for cross-domain clinical prediction.

#### 3.4.1 Decision-level diagnostic analysis for cross-domain transfer

The analysis of the model’s behavior is framed at the decision level, focusing on the transformation of predicted probabilities into binary outputs using a fixed threshold. Let $f_{\theta }(x_{i})\in [0,1]$ be the estimated probability of the positive class for an observation $x_{i}$. The decision rule is defined by applying a fixed threshold τ=0.5 to the predicted probabilities, yielding a binary output ${\hat{y}}_{i}\in \{0,1\}$.

This formulation decouples the probabilistic estimation process from the decision mechanism, establishing a diagnostic strategy to study how the shape of the probability distribution influences the final class assignment.

The diagnostic approach is based on analyzing the conditional distribution of $f_{\theta }(x)$ given the true class y, which allows evaluation of the separability between classes in the probability space. For each evaluation scenario, the predicted probabilities are grouped by the actual label y, enabling characterization of the degree of overlap, concentration, and displacement of the probability mass. This characterization defines the conditions under which the threshold-induced transformation can generate consistent decisions or become unstable across domains.

The application of the threshold τ induces a partitioning of the decision space whose stability depends on the structure and calibration of the probability distribution, event prevalence, and the location of the operating point. Well-separated distributions may still lead to conservative or liberal decisions if the fixed threshold is poorly aligned with the score scale. Conversely, overlapping or shifted distributions can generate dominance of one predicted class, thereby affecting threshold-dependent detection of positive events even when some rank-ordering information remains.

The structural representation of this process is shown in Figure 2, which formalizes the sequence from probabilistic modeling to fixed-threshold classification. The scheme represents in-domain and cross-domain evaluation as empirical checks of probability distributions and operating-point stability rather than as guaranteed decision outcomes. Possible observed behaviors include stable activation, conservative decisions, liberal decisions, or degenerate decisions.

**Figure 2:**
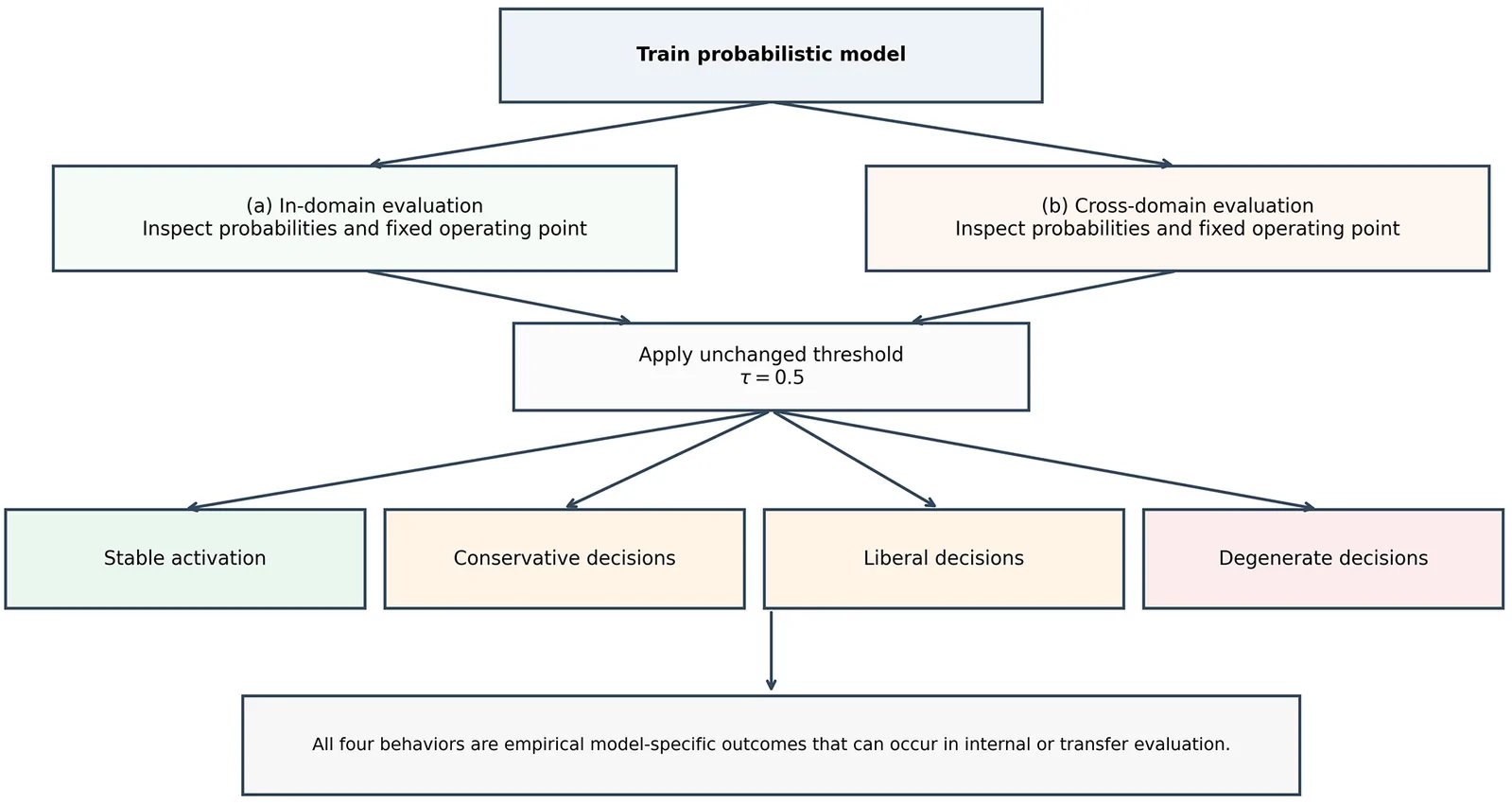
Decision-level diagnostic framework for evaluating probability distributions and fixed operating-point stability.

The decision-level diagnostic analysis is applied consistently across all scenarios defined in the experimental protocol, allowing us to study how variations in the probability distribution translate into changes in classification behavior at the decision level.

## 4 Results

### 4.1 Characterization of the integrated dataset and selection of variables

Table 2 presents the sequential cohort flow used to define the primary 24-h landmark analysis and separates the eICU APACHE-row selection, outcome availability, and landmark-eligibility steps. The primary analyzable cohort contained 110 MIMIC ICU stays from 79 patients and 105 admissions, with 33 in-hospital deaths (30.0%), and 1,324 eICU ICU stays from 1,124 patients and 1,277 hospital-system stays, with 117 in-hospital deaths (8.8%). For eICU, the reduction from raw APACHE rows to the final cohort is therefore not attributed solely to the landmark rule: the APACHE file first contributes 3,676 result rows for 1,838 unique ICU stays, which are reduced to one APACHE result per ICU stay and then filtered for resolvable outcome and 24-h eligibility.

**Table 2:** Sequential cohort flow for the primary 24-h landmark analysis.

Stage	Source/unit	Rows	Unique ICU stays	Deaths
MIMIC outcome-resolved full-episode cohort	MIMIC ICU stays	136	136	46
MIMIC excluded before 24-h landmark	MIMIC ICU stays	26	26	13
MIMIC primary 24-h landmark cohort	MIMIC ICU stays	110	110	33
Local eICU patient-table input	eICU patient table	2,520	2,520	–
Raw APACHE result rows before selection	eICU APACHE rows	3,676	1,838	310
After selecting one APACHE result per ICU stay	eICU ICU stays	1,838	1,838	155
After requiring resolvable hospital mortality outcome	eICU ICU stays	1,838	1,838	155
After 24-h ICU/hospital landmark eligibility	eICU ICU stays	1,324	1,324	117
Unique patients in landmark cohort	Descriptive count	MIMIC: 79; eICU: 1,124	–	–
Admissions/hospital-system stays in landmark cohort	Descriptive count	MIMIC: 105; eICU: 1,277	–	–

The MIMIC landmark cohort includes repeated ICU stays for some patients (110 ICU stays from 79 patients), and the eICU landmark cohort also includes repeated ICU stays (1,324 ICU stays from 1,124 patients). These patient-group counts were used for patient-cluster bootstrap uncertainty intervals in the transfer analysis and for patient-grouped internal validation.

Table 3 summarizes the quantitative effect of replacing the secondary full-episode/full-feature workflow with the corrected primary 24-h shared-feature workflow. The table is not intended to compare two deployable models; it documents how sample size, event counts, discrimination, calibration, and threshold-dependent behavior changed after the landmark restriction, ICU-stay-level MIMIC aggregation, removal of endpoint-derived/source-specific predictors, and strict shared-feature retention.

**Table 3:** Comparison between secondary full-episode workflow and corrected primary 24-h shared-feature workflow.

Element	Secondary full-episode workflow	Corrected primary workflow	Observed change
MIMIC ICU stays	136	110	Landmark eligibility reduced MIMIC by 26 ICU stays
MIMIC deaths	46	33	Landmark cohort has fewer events and lower prevalence (30.0%)
eICU APACHE/full-episode rows	3,676	1,324 ICU stays	corrected primary workflow uses deduplicated outcome-resolved landmark ICU stays
eICU deaths	310	117	eICU prevalence remained low (8.8%)
Feature specification	full-episode/full-feature	24-h strict shared-feature	endpoint-derived and source-specific predictors excluded
LR eICU→MIMIC AUC	0.583	0.598	similar weak discrimination
RF eICU→MIMIC AUC	0.700	0.642	lower corrected AUC; still above 0.5
GB eICU→MIMIC AUC	0.692	0.613	lower corrected AUC
RF eICU→MIMIC positive predictions	0/136	0/110	fixed-threshold suppression persisted
GB eICU→MIMIC positive predictions	0/136	3/110	corrected workflow produced two true positives and one false positive
RF eICU→MIMIC ECE	0.258 (5-bin secondary diagnostic)	0.200 (10-bin primary diagnostic)	calibration remains imperfect; estimators differ by workflow
LR eICU→MIMIC Brier	0.259	0.269	no evidence of improved calibration from correction alone

Regarding variable completeness, the primary retained variable set does not assume that a 29-column structural matrix is equivalent to clinical harmonization. Instead, the primary 24-h model uses 35 pre-encoding predictors derived from 11 shared clinical variable families plus age and gender. Temperature was not retained in the primary shared-feature matrix because eICU missingness was 91.8% after applying the corrected 24-h landmark definition. The secondary 29-variable full-episode matrix is described as a retrospective specification. Core vital-sign and laboratory summaries were present in both sources with low-to-moderate missingness, whereas source-specific fields and insufficiently observed variables were excluded. This pattern indicates that structural column alignment alone is insufficient as evidence of measurement equivalence.

### 4.2 Comparison of distributions between clinical settings

The comparison of clinical variable distributions reveals consistent differences between the MIMIC and eICU datasets in both location and dispersion. For heart rate, both sources exhibit unimodal distributions with a high degree of overlap in the central range; however, a slight shift toward higher values is observed in MIMIC, with its maximum density concentrated around values slightly higher than those observed in eICU. Furthermore, the MIMIC distribution shows a longer right tail, indicating greater variability toward higher values than that of eICU.

Figure 3a presents the comparative distributions of the selected clinical variables in both environments. In Figure 3a, which shows heart rate, both distributions have a similar shape, with an approximately unimodal pattern. However, the MIMIC curve shows a slight shift toward higher values, with greater density in the 85–100 range. eICU, on the other hand, shows a higher concentration in the slightly lower ranges, with a steeper slope of decline following the peak. The overlap between the two curves is high in the mid-range but gradually decreases toward the extreme values, where MIMIC exhibits a longer tail.

**Figure 3:**
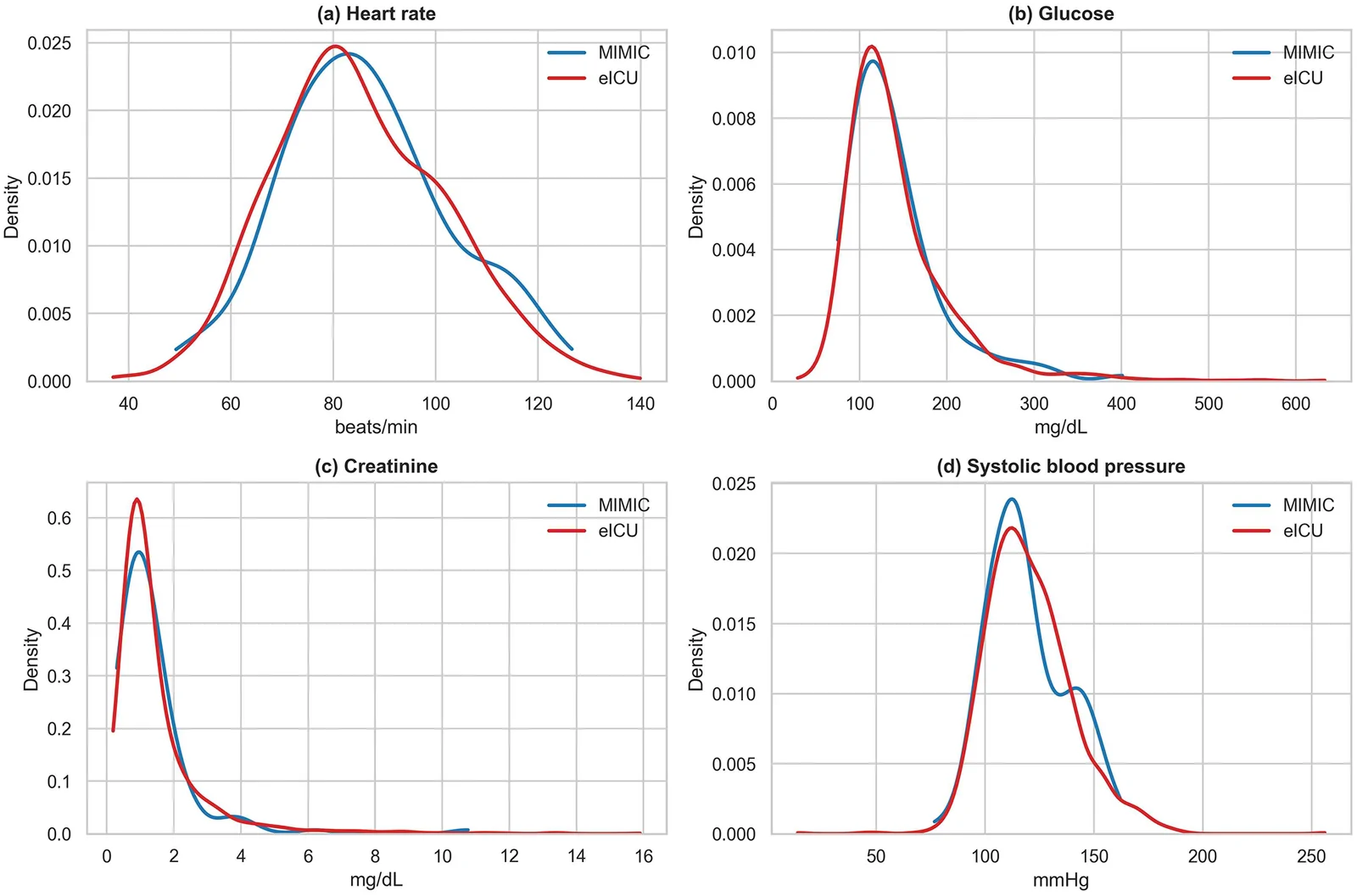
Distribution of key primary shared clinical variables across datasets. **(a)** Heart rate distribution for MIMIC and eICU in beats per minute. **(b)** Glucose level distribution for MIMIC and eICU in mg/dL. **(c)** Creatinine distribution for MIMIC and eICU in mg/dL. **(d)** Systolic blood pressure distribution in mmHg. Kernel-density curves were generated from available non-missing values using the plotting library’s default Gaussian kernel bandwidth.

In Figure 3b, corresponding to glucose levels, the differences between the datasets are more pronounced. eICU shows a more concentrated distribution, with a sharper peak around 110, followed by a rapid drop in density. In contrast, MIMIC shows a broader distribution, with a lower concentration at the peak and a considerable extension toward higher values. The right tail in MIMIC extends well beyond 250, while in eICU, the density decreases more abruptly, indicating greater dispersion in MIMIC.

In Figure 3c, associated with creatinine, a structural difference in the shape of the distributions is observed. eICU shows a high density of values close to zero, with a pronounced peak and a rapid decline. In contrast, MIMIC exhibits a flatter distribution, with lower density at the minimum values and a greater extension toward the upper values. The MIMIC curve maintains a sustained presence over a broader range, reflecting greater variability compared to the strong concentration observed in eICU.

Figure 3d presents the systolic blood-pressure distributions in MIMIC and eICU, completing the comparison using variables included in the primary shared-feature specification. The distributions overlap in the central range but differ in spread and tail behavior, reinforcing that nominally shared variables may still have different empirical distributions across source systems.

### 4.3 Primary 24-h shared-feature performance

The primary analysis used the 24-h landmark cohort and only predictors observed in both MIMIC and eICU. This specification was prioritized over the secondary full-episode/full-feature analysis because it reduces temporal leakage risk and avoids eICU-specific predictors such as predictedhospitalmortality, apachescore, and hospitalid. The performance of the models under intra-environment conditions is presented in Table 4. The first block reports a single stratified 75%/25% eICU holdout split, whereas the integrated block reports five-fold cross-validation across the combined MIMIC-eICU landmark dataset; these estimates should not be confused with the pooled out-of-fold calibration diagnostics reported later in Table 7.

**Table 4:** Primary 24-h shared-feature intra-domain performance under single 75/25 eICU holdout internal validation and integrated cross-validation scenarios.

Scenario	Model	Accuracy	Precision	Recall	F1-score	AUC-ROC
eICU internal validation	LogisticRegression	0.743	0.189	0.586	0.286	0.715
eICU internal validation	RandomForest	0.912	NA	0.000	0.000	0.765
eICU internal validation	GradientBoosting	0.894	0.200	0.069	0.103	0.732
Integrated 5-fold CV	LogisticRegression	0.699±0.031	0.196±0.023	0.600±0.070	0.295±0.032	0.717±0.055
Integrated 5-fold CV	RandomForest	0.895±0.002	0.200±0.400	0.007±0.013	0.013±0.026	0.715±0.032
Integrated 5-fold CV	GradientBoosting	0.883±0.019	0.368±0.267	0.080±0.045	0.127±0.078	0.709±0.062

In the primary 24-h shared-feature internal validation scenario on eICU, apparent performance was much more modest than in the secondary full-episode analysis. Logistic Regression produced the highest recall (0.586) but low precision (0.189), indicating many false-positive activations. Random Forest achieved high accuracy (0.912) and moderate AUC-ROC (0.765), but generated no positive predictions at the fixed 0.5 threshold; precision is therefore reported as NA rather than zero because TP+FP=0. Gradient Boosting showed similar discrimination (AUC-ROC 0.732) but very low recall (0.069).

In integrated five-fold cross-validation, AUC-ROC values remained in the range 0.709–0.717, but threshold-dependent minority-class metrics were weak for tree-based models. Random Forest had recall 0.007±0.013 and F1-score 0.013±0.026, while Gradient Boosting had recall 0.080±0.045 and F1-score 0.127±0.078. Logistic Regression again favored sensitivity over precision, with recall 0.600±0.070 and precision 0.196±0.023. These values indicate that the clinically stricter landmark/shared-feature design substantially reduces the apparent performance observed in the secondary full-episode/full-feature results.

The comparison between internal eICU validation and integrated cross-validation shows that tree-based models retain moderate rank-ordering ability when the evaluation structure allows both domains to contribute to the resampling process. However, this pattern should not be interpreted as evidence of external transportability, because integrated cross-validation still differs from strict train-on-one-domain/test-on-another-domain transfer. Random Forest is especially important: under the primary specification, the fixed 0.5 threshold was already unsuitable in internal eICU validation and remained unsuitable after transfer to MIMIC. Therefore, the absence of positive predictions in MIMIC cannot be attributed exclusively to cross-domain transfer. Fold-level standard deviations were relatively small for accuracy and AUC-ROC, whereas precision and other threshold-dependent minority-class metrics were substantially more variable, particularly for the tree-based models.

### 4.4 Multi-environment evaluation (transfer)

The performance of the models in cross-environment transfer scenarios is presented in Table 5 for the primary 24-h shared-feature specification. In the Train eICU → Test MIMIC scenario, cross-domain discrimination was weak for all models, with AUC-ROC values between 0.598 and 0.642. Threshold-dependent behavior differed substantially across algorithms. Logistic Regression retained partial recall (0.545) but produced 33 false positives among 77 non-events, yielding specificity 0.571. Random Forest generated no positive predictions at τ=0.5, making precision undefined and producing recall and F1-score values of 0.000. Gradient Boosting recovered only minimal positive-class activation, with 2 true positives, 1 false positive, recall 0.061, and F1-score 0.111.

**Table 5:** Primary 24-h shared-feature cross-domain model performance under transfer scenarios.

Scenario	Model	Accuracy	Precision	Recall	F1-score	AUC-ROC
Train eICU → Test MIMIC	Logistic	0.564	0.353	0.545	0.429	0.598
Train eICU → Test MIMIC	RF	0.700	NA	0.000	0.000	0.642
Train eICU → Test MIMIC	GB	0.709	0.667	0.061	0.111	0.613
Train MIMIC → Test eICU	Logistic	0.626	0.094	0.376	0.151	0.539
Train MIMIC → Test eICU	RF	0.888	0.103	0.034	0.051	0.592
Train MIMIC → Test eICU	GB	0.743	0.109	0.265	0.154	0.557

In the reverse scenario, from Train MIMIC → Test eICU, no model achieved clinically strong positive-class detection. Logistic Regression detected 44 of 117 deaths but produced 422 false positives. Random Forest preserved high specificity (0.971) but detected only 4 of 117 deaths. Gradient Boosting detected 31 of 117 deaths but still had low precision (0.109) and F1-score (0.154). The comparison between directions supports a cautious interpretation: the central issue is weak transportable discrimination combined with instability of the probability scale and fixed-threshold decision rule.

Uncertainty in the primary transfer results was quantified using patient-cluster non-parametric bootstrap confidence intervals based on 2,000 resamples within each test set. Each bootstrap replicate sampled patient identifiers with replacement and included all ICU stays belonging to each selected patient, thereby accounting for repeated ICU stays where present. These intervals resample observed target-domain patients while conditioning on the fitted source-domain model; they do not include uncertainty from refitting the source model and do not establish transportability across hospitals. The MIMIC transfer test set contained 79 patient clusters and the eICU transfer test set contained 1,124 patient clusters. Precision is reported as NA when TP+FP=0. In the Train eICU → Test MIMIC scenario, Random Forest had sensitivity 0.000 (95% cluster-bootstrap CI 0.000–0.000), specificity 1.000 (95% cluster-bootstrap CI 1.000–1.000), F1-score 0.000 (95% cluster-bootstrap CI 0.000–0.000), and AUC-ROC 0.642 (95% cluster-bootstrap CI 0.503–0.765). For this all-negative Random Forest prediction pattern, the sensitivity, specificity, and F1 intervals are degenerate bootstrap intervals: resampling the observed target-patient predictions cannot create positive classifications, so these intervals describe invariance of the observed prediction pattern under target-patient resampling rather than absence of population-level or model-fitting uncertainty. Gradient Boosting had sensitivity 0.061 (95% cluster-bootstrap CI 0.000–0.143), specificity 0.987 (95% cluster-bootstrap CI 0.956–1.000), F1-score 0.111 (95% cluster-bootstrap CI 0.000–0.242), and AUC-ROC 0.613 (95% cluster-bootstrap CI 0.461–0.755). Logistic Regression had sensitivity 0.545 (95% cluster-bootstrap CI 0.345–0.735), specificity 0.571 (95% cluster-bootstrap CI 0.424–0.687), F1-score 0.429 (95% cluster-bootstrap CI 0.260–0.575), and AUC-ROC 0.598 (95% cluster-bootstrap CI 0.459–0.720). These uncertainty results are summarized in Table 6.

**Table 6:** Uncertainty summary for primary 24-h shared-feature cross-domain transfer results.

Scen.	Model	Precision	Sensitivity	Specificity	F1-score	AUC-ROC	Brier
E2M	LR	0.353	0.545 [0.345, 0.735]	0.571 [0.424, 0.687]	0.429 [0.260, 0.575]	0.598 [0.459, 0.720]	0.269 [0.224, 0.322]
E2M	RF	NA	0.000 [0.000, 0.000]	1.000 [1.000, 1.000]	0.000 [0.000, 0.000]	0.642 [0.503, 0.765]	0.241 [0.164, 0.333]
E2M	GB	0.667	0.061 [0.000, 0.143]	0.987 [0.956, 1.000]	0.111 [0.000, 0.242]	0.613 [0.461, 0.755]	0.242 [0.164, 0.333]
M2E	LR	0.094	0.376 [0.291, 0.466]	0.650 [0.622, 0.678]	0.151 [0.111, 0.192]	0.539 [0.483, 0.596]	0.244 [0.230, 0.259]
M2E	RF	0.103	0.034 [0.008, 0.072]	0.971 [0.962, 0.981]	0.051 [0.012, 0.106]	0.592 [0.537, 0.642]	0.130 [0.123, 0.138]
M2E	GB	0.109	0.265 [0.183, 0.352]	0.790 [0.766, 0.815]	0.154 [0.105, 0.204]	0.557 [0.498, 0.612]	0.186 [0.170, 0.202]

E2M denotes Train eICU → Test MIMIC; M2E denotes Train MIMIC → Test eICU. Bracketed intervals are patient-cluster bootstrap 95% confidence intervals from 2,000 resamples using patient_group_id as the target-domain cluster unit while conditioning on the fitted source model. Precision is reported as NA when TP+FP=0. The all-negative RF E2M sensitivity, specificity, and F1 intervals are degenerate bootstrap intervals caused by invariant observed predictions under resampling.

Table 7 compares source-domain and transfer behavior for the primary 24-h shared-feature analysis using threshold-dependent, discrimination, precision-recall, and calibration summaries. The event prevalence was 0.088 in eICU and 0.300 in the MIMIC landmark cohort, so average precision must be interpreted relative to these baselines. In eICU internal out-of-fold validation, Random Forest had AUC-ROC 0.694 and average precision 0.162 but recall 0.009 and F1-score 0.017 at τ=0.5, confirming that its fixed-threshold failure was already present before transfer. After eICU-to-MIMIC transfer, its AUC-ROC was 0.642 and average precision was 0.438, but the operating point still produced no positive predictions. Logistic Regression showed a different decision pathology: eICU-to-MIMIC transfer retained partial sensitivity but with substantial false-positive burden and poor calibration (Brier 0.269; ECE 0.235). Gradient Boosting showed very limited positive-class activation in transfer despite nonzero average precision.

**Table 7:** Primary 24-h shared-feature source-domain and transfer diagnostics.

Scenario	Model	Recall	Specificity	F1	AUC	AP	Brier	ECE	Intercept/slope
eICU internal	LR	0.556	0.716	0.248	0.708	0.251	0.198	0.300	−2.250/0.585
eICU internal	RF	0.009	0.998	0.017	0.694	0.162	0.079	0.026	−0.765/0.618
eICU internal	GB	0.060	0.982	0.096	0.742	0.209	0.081	0.037	−0.700/0.588
E2M transfer	LR	0.545	0.571	0.429	0.598	0.402	0.269	0.235	−0.848/0.255
E2M transfer	RF	0.000	1.000	0.000	0.642	0.438	0.241	0.200	−0.620/0.086
E2M transfer	GB	0.061	0.987	0.111	0.613	0.459	0.242	0.227	0.163/0.371
MIMIC internal	LR	0.394	0.597	0.338	0.503	0.401	0.289	0.251	−0.821/0.107
MIMIC internal	RF	0.061	0.948	0.103	0.535	0.338	0.217	0.098	−0.583/0.282
MIMIC internal	GB	0.182	0.870	0.245	0.603	0.392	0.254	0.202	−0.533/0.165
M2E transfer	LR	0.376	0.650	0.151	0.539	0.123	0.244	0.323	−2.293/0.113
M2E transfer	RF	0.034	0.971	0.051	0.592	0.117	0.130	0.211	−1.792/0.653
M2E transfer	GB	0.265	0.790	0.154	0.557	0.108	0.186	0.206	−2.204/0.088

AP denotes average precision; ECE denotes 10-bin expected calibration error; calibration intercept and slope are from post hoc logistic recalibration. E2M denotes Train eICU → Test MIMIC; M2E denotes Train MIMIC → Test eICU. Internal rows are pooled out-of-fold estimates.

Because the MIMIC source cohort is small (110 landmark ICU episodes with 33 deaths), internal MIMIC validation was treated as an unstable diagnostic rather than as definitive evidence of source-domain performance. In 20 repeats of stratified five-fold cross-validation, AUC-ROC means were 0.474 for Logistic Regression, 0.585 for Random Forest, and 0.553 for Gradient Boosting, with fold-level standard deviations of 0.137, 0.109, and 0.118, respectively. Random Forest produced zero positive predictions in 22 of 100 folds and Gradient Boosting in 1 of 100 folds, whereas Logistic Regression did not produce zero-positive folds. Patient-grouped five-fold internal validation further reduced apparent MIMIC performance and showed weak threshold-dependent behavior across models (Table 8). This analysis indicates that the MIMIC-to-eICU transfer results should be interpreted against a weak and unstable MIMIC source model, not as a clean demonstration of degradation from a strong source-domain baseline.

**Table 8:** Patient-grouped five-fold internal validation for the primary 24-h shared-feature cohort.

Source	Model	Recall	Specificity	F1	AUC	AP	Brier	ECE	TP/FP/FN/TN
eICU	LR	0.607	0.714	0.266	0.708	0.242	0.196	0.297	71/345/46/862
eICU	RF	0.000	0.998	0.000	0.681	0.165	0.079	0.021	0/2/117/1205
eICU	GB	0.103	0.986	0.164	0.739	0.231	0.078	0.033	12/17/105/1190
MIMIC	LR	0.303	0.494	0.244	0.365	0.279	0.392	0.390	10/39/23/38
MIMIC	RF	0.030	0.870	0.045	0.476	0.308	0.233	0.170	1/10/32/67
MIMIC	GB	0.152	0.792	0.185	0.483	0.278	0.333	0.325	5/16/28/61

As a secondary diagnostic for the secondary full-episode/full-feature specification, calibration and post hoc threshold sensitivity were also evaluated using the earlier five-quantile-bin diagnostic retained only for comparison with the secondary workflow. This analysis is not treated as an operational threshold-selection procedure because the F1-maximizing thresholds were selected using the labeled test sets; it is therefore an oracle/post hoc diagnostic used only to assess whether ranking information remained below the fixed conventional 0.5 threshold. In Train eICU → Test MIMIC, average precision was 0.541 for Random Forest and 0.559 for Gradient Boosting, confirming that some ranking information remained despite zero positive predictions at τ=0.5. Calibration gaps were nevertheless large: the secondary five-bin ECE was 0.258 for Random Forest, 0.285 for Gradient Boosting, and 0.183 for Logistic Regression. Calibration intercept/slope were 1.964/1.030 for Random Forest, 1.886/0.802 for Gradient Boosting, and −0.542/0.082 for Logistic Regression. Post hoc F1-maximizing thresholds were much lower than 0.5 for the tree-based models (0.067 for Random Forest and 0.027 for Gradient Boosting), recovering recall values of 0.783 and 0.826, respectively, but with only moderate precision (0.480 and 0.469) and specificity near 0.56 and 0.52. These results indicate that the transferred models were not devoid of all ordering information; rather, their probability scale was poorly aligned with the fixed conventional threshold.

The full-feature 24-h landmark sensitivity analysis, which retained variables when structurally present in the broader feature table, is shown in Table 9. Its results were similar to the primary shared-feature landmark analysis for Random Forest and Gradient Boosting, but Logistic Regression became more sensitive in eICU-to-MIMIC transfer (24 true positives, recall 0.727) at the cost of lower specificity (36 true negatives and 41 false positives). Gradient Boosting produced four positive predictions in this sensitivity analysis, comprising three true positives and one false positive. Random Forest generated no positive predictions in the eICU-to-MIMIC direction, so its precision is reported as NA rather than zero. This supports a cautious interpretation that fixed-threshold behavior is sensitive to feature-alignment decisions, whereas Random Forest repeatedly shows a highly conservative fixed-threshold operating point that is visible before and after transfer.

**Table 9:** Secondary full-feature 24-h landmark sensitivity analysis for cross-domain transfer.

Scenario	Model	Accuracy	Precision	Recall	F1-score	AUC-ROC
E2M	Logistic	0.545	0.369	0.727	0.490	0.590
E2M	RF	0.700	NA	0.000	0.000	0.631
E2M	GB	0.718	0.750	0.091	0.162	0.628
M2E	Logistic	0.662	0.107	0.385	0.168	0.539
M2E	RF	0.888	0.103	0.034	0.051	0.621
M2E	GB	0.792	0.137	0.256	0.179	0.580

E2M denotes Train eICU → Test MIMIC; M2E denotes Train MIMIC → Test eICU. Precision is reported as NA when TP+FP=0.

The full-episode strict shared-feature sensitivity analysis further clarifies the role of variable equivalence (Table 10). In Train eICU → Test MIMIC, Random Forest still generated no true positives after excluding eICU-specific and source-absent predictors (sensitivity 0.000, F1-score 0.000, AUC-ROC 0.692). Logistic Regression improved its sensitivity from 0.348 to 0.500, with F1-score increasing from 0.381 to 0.455. Gradient Boosting no longer produced a complete fixed-threshold collapse under this restriction, recovering 12 true positives (sensitivity 0.261, precision 0.667, F1-score 0.375, AUC-ROC 0.750). Therefore, the Random Forest transfer failure is more robust to removal of non-shared variables, whereas the Gradient Boosting result is sensitive to broader feature-alignment assumptions.

**Table 10:** Secondary full-episode cross-domain transfer sensitivity restricted to predictors observed in both MIMIC and eICU.

Scenario	Model	Accuracy	Precision	Recall	F1-score	AUC-ROC
E2M	Logistic	0.596	0.418	0.500	0.455	0.593
E2M	RF	0.654	0.000	0.000	0.000	0.692
E2M	GB	0.706	0.667	0.261	0.375	0.750
M2E	Logistic	0.754	0.140	0.374	0.204	0.694
M2E	RF	0.887	0.268	0.194	0.225	0.661
M2E	GB	0.808	0.133	0.232	0.169	0.617

E2M denotes Train eICU → Test MIMIC; M2E denotes Train MIMIC → Test eICU.

### 4.5 Analysis of the model’s behavior

Figure 4 presents the predicted probability distributions for the primary 24-h shared-feature transfer analysis. In Figure 4a, corresponding to Logistic Regression in the Train eICU → Test MIMIC scenario, the survival and death probability distributions overlap substantially around the fixed threshold. This explains why Logistic Regression retained partial sensitivity but also produced substantial false-positive burden in Table 5.

**Figure 4:**
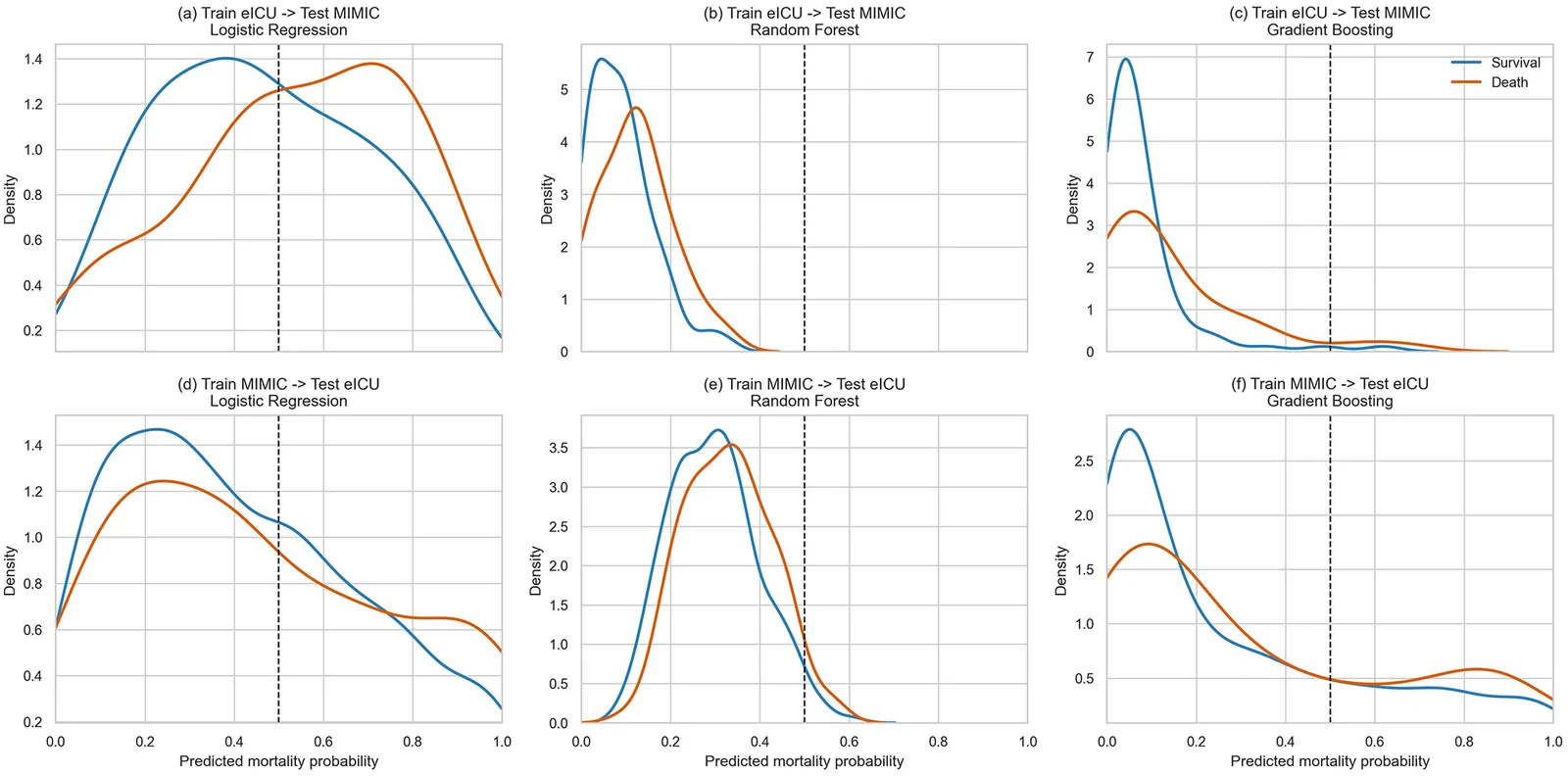
Predicted probability distributions in the primary 24-h shared-feature cross-domain transfer analysis. **(a)**–**(c)** Train eICU → Test MIMIC and **(d)**–**(f)** Train MIMIC → Test eICU for Logistic Regression, Random Forest, and Gradient Boosting. The dashed vertical line indicates the fixed conventional threshold τ=0.5.

In Figure 4b, Random Forest probabilities remain below τ=0.5 for all MIMIC test cases, which produces a constant negative classification despite an AUC-ROC above 0.5. In Figure 4c, Gradient Boosting shows a less extreme pattern: a small subset of MIMIC cases crosses the threshold, producing limited recovery of positive-class activation.

In the reverse Train MIMIC → Test eICU direction, Logistic Regression again produces a broad probability distribution with many values above the threshold, whereas Random Forest remains highly conservative and Gradient Boosting produces an intermediate pattern. Across both directions, the figure shows that transfer failure is not adequately summarized by AUC-ROC alone; the location of the probability distribution relative to the fixed threshold determines whether mortality cases are actually activated as positive decisions.

The analysis of Table 11 quantifies these behavioral patterns in terms of classification decisions. In the Train eICU → Test MIMIC scenario, Logistic Regression produced TP=18 and FP=33, Random Forest produced TP=0 and FP=0, and Gradient Boosting produced TP=2 and FP=1. Therefore, the same transfer direction can fail in different operational ways: false-positive burden for Logistic Regression, complete positive-class suppression for Random Forest, and very limited activation for Gradient Boosting.

**Table 11:** Confusion matrix summary and classification performance in the primary 24-h shared-feature cross-domain transfer scenarios.

Scenario	Model	TN	FP	FN	TP	Sensitivity	Specificity
Train eICU → Test MIMIC	Logistic	44	33	15	18	0.545	0.571
Train eICU → Test MIMIC	RF	77	0	33	0	0.000	1.000
Train eICU → Test MIMIC	GB	76	1	31	2	0.061	0.987
Train MIMIC → Test eICU	Logistic	785	422	73	44	0.376	0.650
Train MIMIC → Test eICU	RF	1,172	35	113	4	0.034	0.971
Train MIMIC → Test eICU	GB	953	254	86	31	0.265	0.790

In the Train MIMIC → Test eICU scenario, the confusion matrix structure reflects a distinct interaction between the probability distribution and the decision threshold. Logistic Regression generated many false positives (FP=422), while Random Forest generated very few positive predictions and missed 113 of 117 mortality cases. Gradient Boosting showed intermediate behavior but still missed most events. These results reinforce that fixed-threshold transportability is fragile even when models preserve some apparent discrimination.

Figure 5 shows calibration curves for the primary 24-h shared-feature analysis using the same 10 equal-width ECE definition reported in Table 7. The curves show visible departures from the ideal diagonal in both internal and transfer settings. In the eICU-to-MIMIC transfer panel, Random Forest and Gradient Boosting assign most cases to low predicted-probability ranges while observed event rates remain high in several bins, explaining how nonzero rank-ordering can coexist with poor fixed-threshold activation.

**Figure 5:**
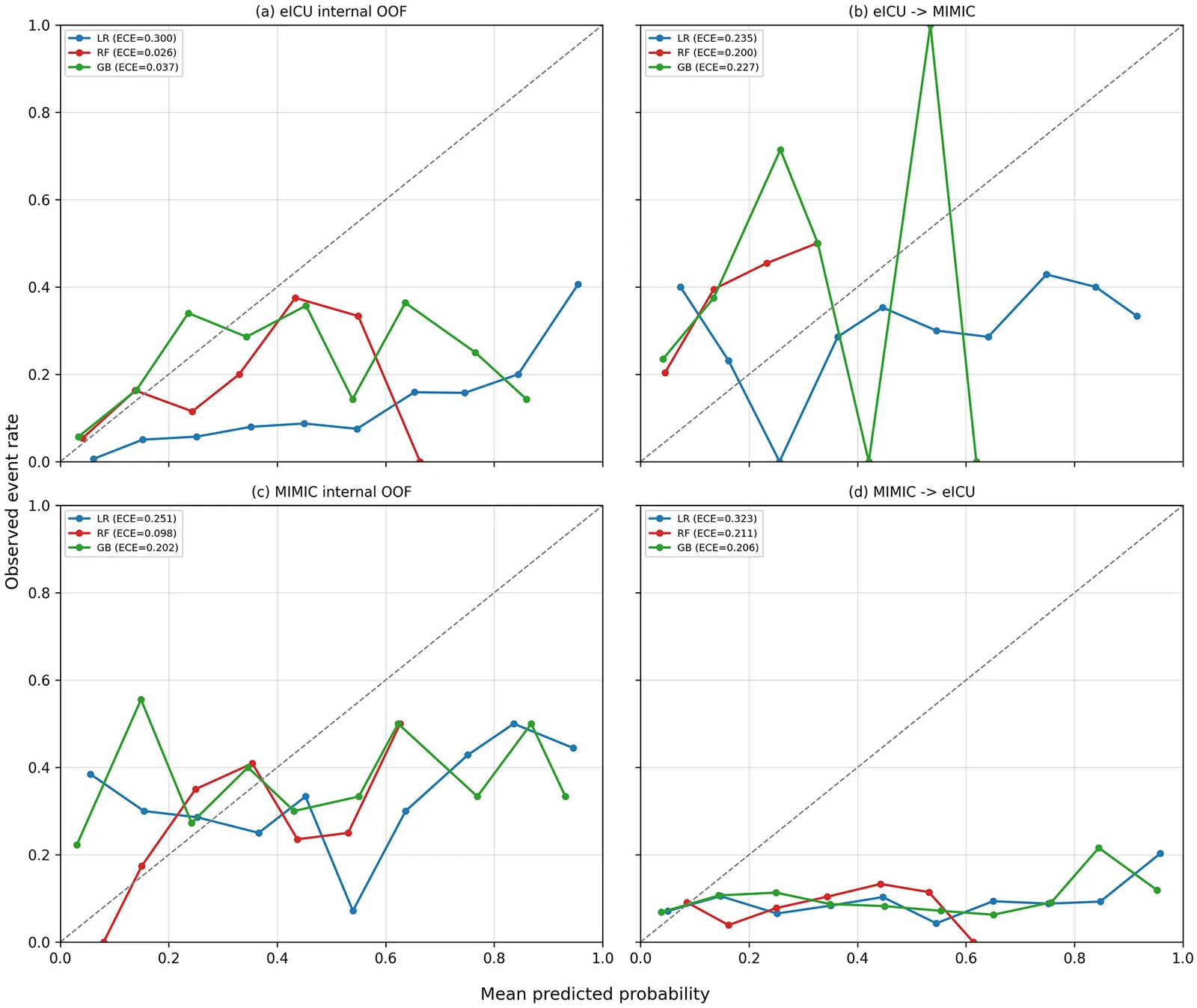
Calibration curves for the primary 24-h shared-feature analysis. **(a)** eICU internal out-of-fold validation. **(b)** Train eICU → Test MIMIC. **(c)** MIMIC internal out-of-fold validation. **(d)** Train MIMIC → Test eICU. Points represent non-empty equal-width probability bins; the dashed line indicates perfect calibration.

## 5 Discussion

The results show that the full-episode/full-feature performance estimates were sensitive to both temporal design and feature-equivalence assumptions. Under the primary 24-h shared-feature specification, cross-domain AUC-ROC values were weak and, in most comparisons, statistically compatible with chance-level performance. Threshold-dependent minority-class detection remained clinically fragile. This pattern aligns with prior literature on dataset shift and generalization in clinical settings and supports a cautious interpretation of the observed transfer behavior. In particular, the degradation observed in transfer scenarios is consistent with studies demonstrating the sensitivity of models to variations in the distribution of populations and clinical centers (7, 8). The concentration of probabilities relative to the decision threshold reflects threshold-specific instability of positive-class activation rather than complete loss of discrimination, previously associated with differences in event prevalence and in the distributions of clinical variables (9, 10). The primary and secondary analyses support a model-specific interpretation: Random Forest remained highly conservative and failed to generate positive predictions in eICU-to-MIMIC transfer under the primary specification, but this was a persistence and amplification of an internal fixed-threshold failure rather than a failure induced only by the target domain; Gradient Boosting recovered limited positive-class detection once the analysis was restricted to shared early-window variables. This phenomenon has also been linked to the instability of selected feature sets when transferred between domains, which directly affects model consistency (11). Furthermore, the limited ability of models to maintain sensitivity in unobserved environments aligns with observations from multicenter studies, in which external validation reveals substantial performance drops relative to internal validation (14). Additionally, recent research has highlighted that the absence of explicit calibration and the use of fixed thresholds can amplify the effects of class imbalance and overlap, impacting the activation of the positive class (4, 12, 13).

Methodologically, the decision-level diagnostic analysis allows model behavior to be inspected beyond aggregated metrics by explicitly examining the relationship between the probability distribution $f_{\theta }(x)$ and the decision threshold τ. The contribution is not that probability distributions and thresholds are novel in themselves, but that their joint inspection exposes an operational failure mode that aggregate discrimination metrics alone can obscure under internal validation and cross-domain transfer. This formulation enables identification of fixed-threshold positive-class activation failure not evident solely from metrics such as AUC-ROC or accuracy. In particular, the use of decision-level diagnostics allows interpretation of sensitivity loss as a consequence of the probability distribution structure relative to the fixed threshold, rather than attributing it exclusively to the model’s ranking capacity.

Comparative analysis between models shows that no algorithm provided robust cross-domain mortality detection under the primary design. Random Forest was the most conservative model and repeatedly failed to activate the positive class in the eICU-to-MIMIC direction. Gradient Boosting was more sensitive to feature specification: it collapsed in the secondary full-feature analysis but recovered partial sensitivity under the primary 24-h shared-feature design. Logistic Regression preserved sensitivity in some transfer settings, but often by producing many false positives. This result suggests that model robustness cannot be inferred from internal AUC-ROC alone and must be evaluated jointly with calibration, threshold behavior, and clinically meaningful error profiles.

The main contribution of this work lies in integrating a multi-environment evaluation protocol with decision-level diagnostic analysis. Unlike approaches focused only on global metrics, this study identifies conditions under which a fixed conventional threshold lacks operational meaning, thereby linking the shape of the predicted-probability distribution to the structure of the confusion matrix. This approach is particularly relevant in clinical applications, where error interpretation and the ability to detect positive events are more critical than average performance.

However, the results have limitations that should be considered when interpreting the findings. First, using a fixed threshold (τ=0.5) imposes a constraint that may be suboptimal in scenarios with strong class imbalance. Although this decision allows standardized comparison between models, it can also accentuate the loss of sensitivity in skewed distributions. The bootstrap, precision-recall, Brier-score, calibration-gap, and threshold-sensitivity analyses show that both sampling uncertainty and imperfect calibration are material, particularly in the eICU-to-MIMIC transfer where the MIMIC test set is small. Second, the construction of the integrated dataset assumes structural alignment of variables across domains, which may mask semantic or clinical differences in their measurement. This is not a merely theoretical limitation: the shared-feature sensitivity analysis showed that removing source-specific or source-absent predictors changed Gradient Boosting behavior. This assumption may affect the model’s validity in contexts where data acquisition protocols differ substantially.

Additionally, the evaluation is based on retrospective datasets, which limits the generalizability of the results to prospective or real-time scenarios. The primary 24-h landmark design reduces, but does not eliminate, temporal leakage risk because it remains retrospective and depends on the timestamp fidelity of the source databases. Calibration was assessed through summary measures and post hoc recalibration diagnostics; prospective calibration, calibration-in-the-large estimated in an independent deployment cohort, and clinically selected decision thresholds remain outside the scope of this study. Although the analysis focuses on classic machine learning models, it does not explore more complex architectures that might exhibit different behaviors under transfer conditions. These limitations suggest that while the proposed approach enables identification of relevant patterns in model behavior, its application should account for specific adjustments tailored to the clinical domain, class balance, and the availability of representative data, especially in scenarios where decision-making depends on accurate detection of rare events.

Finally, this study does not perform hospital-level leave-site-out validation within eICU, and the MIMIC demo cohort is too small to support definitive population-level claims. Patient-grouped internal validation was conducted as a sensitivity analysis. Cross-domain uncertainty intervals were estimated through patient-cluster bootstrap resampling, in which target-domain patients were sampled with replacement and all ICU stays belonging to each selected patient were retained. These intervals condition on the fitted source-domain model and therefore do not incorporate uncertainty arising from refitting the source model or establish transportability across hospitals. The findings should therefore be interpreted as exploratory evidence consistent with threshold-specific instability under dataset shift rather than as a definitive estimate of deployment performance or as identification of a unique causal mechanism. Future work should use the full MIMIC and eICU resources, prespecified landmark times, site-aware validation, source-model refitting uncertainty, and reporting aligned with clinical prediction-model guidance such as TRIPOD-AI and PROBAST-AI.

## 6 Conclusions

In these selected 24-h landmark cohorts, the evaluated clinical prediction models could not be interpreted adequately from aggregate discrimination metrics alone. The primary evidence comes from a 24-h landmark analysis restricted to variables observed with sufficient completeness in both MIMIC and eICU. Under this stricter design, cross-domain discrimination was weak and threshold-dependent mortality detection was unstable across all models. Random Forest showed the strongest positive-class suppression in eICU-to-MIMIC transfer, but this fixed-threshold failure was already present during internal eICU validation. Gradient Boosting detected only two MIMIC deaths in eICU-to-MIMIC transfer, and Logistic Regression retained only partial sensitivity with substantial false-positive burden.

Logistic Regression maintained nonzero sensitivity in both transfer directions, but this did not constitute robust transportability. In the eICU-to-MIMIC direction, it detected 18 of 33 deaths but misclassified 33 of 77 non-events as deaths. In the reverse direction, it detected 44 of 117 deaths but generated 422 false positives. The study did not define clinical utility thresholds or evaluate net benefit, so these findings should be interpreted as unfavorable fixed-threshold operating behavior rather than as a formal clinical-utility assessment.

The observed probability distributions were associated with the behavior of the fixed decision threshold τ. When the probability mass was concentrated in regions below the threshold, the classification process converged toward constant negative decisions, regardless of the actual class. This pattern explains the simultaneous presence of high specificity and the absence of positive-class detection in certain scenarios, which cannot be captured by metrics such as accuracy or AUC-ROC in isolation.

This work uses a multi-environment evaluation scheme that distinguishes between intra-domain performance and transfer behavior, and a decision-level diagnostic analysis that explicitly links probabilistic estimation to the final decision. This integration enables identification of specific conditions under which a model retains partial ranking ability but fails to activate the positive class under an unchanged fixed threshold.

The results are consistent with the view that integrating heterogeneous clinical datasets introduces challenges related to variable alignment and distributional consistency. The difference in size, structure, and distribution between MIMIC and eICU was associated with misalignment patterns affecting model behavior, even when common variables were used. For this reason, future external-validation studies should privilege clinically harmonized variable dictionaries over purely structural column alignment.

Future work should evaluate probabilistic calibration strategies and thresholds selected only from source-domain validation data or from prespecified clinical-utility criteria before external testing. It is also relevant to explore learning methods that explicitly integrate information from multiple domains during training while preserving a locked external-test protocol. Extending the analysis to larger complete datasets, cluster-aware validation designs, and prospective scenarios will allow a more reliable evaluation of generalizability.

## Data Availability

Publicly available datasets were analyzed in this study. This data can be found here: The datasets analyzed in this study are publicly available through PhysioNet (https://physionet.org/), including the MIMIC and eICU databases. Access requires credentialing and approval due to data use agreements. No accession numbers are applicable.
